# Farnesoid X Receptor (FXR) Activation and FXR Genetic Variation in Inflammatory Bowel Disease

**DOI:** 10.1371/journal.pone.0023745

**Published:** 2011-08-22

**Authors:** Rian M. Nijmeijer, Raffaella M. Gadaleta, Saskia W. C. van Mil, Adriaan A. van Bodegraven, J. Bart A. Crusius, Gerard Dijkstra, Daan W. Hommes, Dirk J. de Jong, Pieter C. F. Stokkers, Hein W. Verspaget, Rinse K. Weersma, C. Janneke van der Woude, Janneke M. Stapelbroek, Marguerite E. I. Schipper, Cisca Wijmenga, Karel J. van Erpecum, Bas Oldenburg, on behalf of the Dutch Initiative on Crohn, Colitis (ICC)

**Affiliations:** 1 Department of Surgery, University Medical Center Utrecht, Utrecht, The Netherlands; 2 Department of Gastroenterology and Hepatology, University Medical Center Utrecht, Utrecht, The Netherlands; 3 Department of Metabolic and Endocrine Diseases, University Medical Center Utrecht and Netherlands Metabolomics Centre, Utrecht, The Netherlands; 4 Laboratory of Lipid Metabolism and Cancer, Consorzio Mario Negri Sud, Santa Maria Imbaro (Ch), Italy; 5 Department of Gastroenterology and Hepatology, VU University Medical Center, Amsterdam, The Netherlands; 6 Laboratory of Immunogenetics, Department of Pathology, VU University Medical Center, Amsterdam, The Netherlands; 7 Department of Gastroenterology and Hepatology, University Medical Center Groningen, Groningen, The Netherlands; 8 Department of Gastroenterology and Hepatology, Leiden University Medical Center, Leiden, The Netherlands; 9 Department of Gastroenterology and Hepatology, Radboud University Nijmegen Medical Centre, Nijmegen, The Netherlands; 10 Department of Gastroenterology and Hepatology, Amsterdam Medical Center, Amsterdam, The Netherlands; 11 Department of Gastroenterology and Hepatology, Erasmus Medical Center, Rotterdam, The Netherlands; 12 Department of Pathology, University Medical Center Utrecht, Utrecht, The Netherlands; 13 Department of Genetics, University Medical Center Groningen, Groningen, The Netherlands; Charité-University Medicine Berlin, Germany

## Abstract

**Background:**

We previously showed that activation of the bile salt nuclear receptor Farnesoid X Receptor (FXR) protects against intestinal inflammation in mice. Reciprocally, these inflammatory mediators may decrease FXR activation. We investigated whether FXR activation is repressed in the ileum and colon of inflammatory bowel disease (IBD) patients in remission. Additionally, we evaluated whether genetic variation in *FXR* is associated with IBD.

**Methods:**

mRNA expression of *FXR* and FXR target gene *SHP* was determined in ileal and colonic biopsies of patients with Crohn's colitis (n = 15) and ulcerative colitis (UC; n = 12), all in clinical remission, and healthy controls (n = 17). Seven common tagging SNPs and two functional SNPs in *FXR* were genotyped in 2355 Dutch IBD patients (1162 Crohn's disease (CD) and 1193 UC) and in 853 healthy controls.

**Results:**

mRNA expression of *SHP* in the ileum is reduced in patients with Crohn's colitis but not in patients with UC compared to controls. mRNA expression of villus marker *Villin* was correlated with *FXR* and *SHP* in healthy controls, a correlation that was weaker in UC patients and absent in CD patients. None of the SNPs was associated with IBD, UC or CD, nor with clinical subgroups of CD.

**Conclusions:**

FXR activation in the ileum is decreased in patients with Crohn's colitis. This may be secondary to altered enterohepatic circulation of bile salts or transrepression by inflammatory signals but does not seem to be caused by the studied SNPs in *FXR*. Increasing FXR activity by synthetic FXR agonists may have benefit in CD patients.

## Introduction

Inflammatory bowel disease (IBD) may lead to potentially severe complications and even mortality [1]. Although the exact etiology is unclear, it is thought to result from a combination of mucosal immune system dysregulation, hyper-reactivity against the intestinal microbiota, and a compromised intestinal epithelial barrier function in genetically predisposed individuals [2]. Genes associated with IBD highlight key pathogenic mechanisms, including disturbed anti-bacterial defense (e.g. *NOD2*, *ATG16L1*, cathelicidin, defensins) and barrier function (e.g. *PARD3*, *MAGI2*, myosin IXB) [3]-[8]. In recent genome-wide association studies, the total number of susceptibility loci amounts to 99, but this probably accounts for only 16% of the ulcerative colitis (UC) [9] and 20% of the Crohn's disease (CD) heritability [10]. It has been estimated that future genome-wide association scans will only yield a few more percent of CD and UC heritability. Biological pathway-based analyses or studies focusing on genes involved in established or plausible pathogenetic pathways may be an alternative approach [11].

The bile salt nuclear Farnesoid X Receptor (NR1H4, nuclear receptor subfamily 1, group H, member 4, alias FXR on chromosome 12q23.1) is a member of the superfamily of nuclear receptors. Nuclear receptors are ligand-activated transcription factors that, in response to lipophilic ligands (e.g. hormones, vitamins and dietary lipids), regulate many aspects of mammalian physiology, including development, reproduction and metabolism [12], [13]. *FXR* is mainly expressed in the ileum and liver. Upon activation by bile salts, FXR binds as a heterodimer with Retinoid X Receptor to the FXR responsive elements on the promoters of target genes, such as the small heterodimer partner (SHP). Via this classical route of transactivation, FXR regulates transcription of genes involved in bile salt synthesis, transport and metabolism in the liver and intestine [14]. FXR has also been implicated in immune modulation and barrier function in the intestine [15], [16]. We recently reported that pharmacological FXR activation decreases the severity of inflammation and preserves the intestinal barrier integrity in two well-established murine colitis models [17]. As already described for other nuclear receptors, the mechanism by which FXR modulates inflammation is most probably through transrepression of nuclear transcription factor kappa B (NF-κB) signaling. Dysregulated activation of NF-κB has previously been identified as a key factor in the pro-inflammatory response in IBD, resulting in strongly enhanced expression of pro-inflammatory genes such as Tumor Necrosis Factor α or Interleukin-1β and recruitment of an excess of inflammatory cells to the intestinal wall [18]. Notably, we and others previously showed that there is reciprocal repression of FXR and NF-κB *in vitro* and *in vivo* [19], [20].

We therefore investigated *FXR* and FXR target gene mRNA expression in patients with CD and UC in clinical remission. In addition, since FXR acts as a regulator of intestinal inflammation, we hypothesized that polymorphisms in *FXR* might be associated with IBD and tested this hypothesis in a large Dutch cohort of IBD patients and controls.

## Materials and Methods

### Patients in mRNA expression study

Seventeen healthy subjects (male/female 7/10; age 55±12.1 years), 15 patients with Crohn's colitis (male/female 5/10; age 46±9.8 years) and 12 patients with UC (male/females 4/8; age 44±9.8 years) were enrolled in this study. Montreal classification and medication at the time of endoscopy are shown in Table 1. All IBD patients were in clinical and endoscopic remission without significant histological activity. Patients with significant endoscopic or histological disease activity were excluded. The indication for colonoscopy was screening for cancer or polyps in healthy controls and scheduled dysplasia screening in IBD patients. Biopsies were obtained from the ileum and ascending colon, immediately frozen in liquid nitrogen and subsequently stored at −80°C, until further processing. Written informed consent was obtained from all subjects and the study was approved by the Medical Ethical Committee of the University Medical Centre Utrecht.

**Table 1:** Montreal classification and medication of patients of the mRNA expression study.

Characteristic	Healthy subjects	CD patients	UC patients
Number	17	15	12
Male gender (%)	7 (41%)	5 (33%)	4 (33%)
Disease localization (Montreal classification) for CD			
L1: ileum		0	
L2: colon		8 (53%)	
L3: ileocolonic		7 (47%)	
L4: upper disease		2 (13%)	
Disease behavior (Montreal classification for CD			
B1: nonstricturing, nonpenetrating		13 (87%)	
B2: stricturing		1 (7%)	
B3: penetrating		1 (7%)	
P: perianal disease		1 (7%)	
Disease localization (Montreal classification) for UC			
E1: ulcerative proctitis			0
E2: left-sided (distal) UC			7 (58%)
E3: extensive UC (pancolitis)			5 (42%)
Medication			
Steroids		2 (13%)	1 (8%)
Mesalamine		8 (53%)	12 (100%)
Thioguanines		4 (27%)	4 (33%)
Methotrexate		2 (13%)	0
Anti-TNF agents		1 (7%)	0

### mRNA extraction and qRT-PCR analysis

Human biopsies of ileum and ascending colon were homogenized (Omni TH tissue homogenizer, Omni International, Kennesaw, USA) and RNA was isolated using RNeasy Micro kit (Qiagen GmbH, Hilden, Germany) according to the manufacturer's instructions. The quantity, quality and integrity of isolated mRNA were confirmed by absorption measurement and RNA gel electrophoresis. Subsequently, cDNA was generated from 500 ng of total RNA using SuperScript II Reverse Transcriptase (Invitrogen, Carlsbad, CA, USA) and random hexamers (Roche, Basel, Switzerland). qRT-PCR analysis was carried out using SYBR green PCR master mix (Biorad, Veenendaal, The Netherlands) and a MyIQ real time PCR cycler (Biorad). Values were quantified using the comparative threshold cycle method. *FXR* and its target genes are exclusively expressed in the differentiated enterocyte on the top of the villi [15], [17], [19]. In order to estimate the distribution between villi and crypts in the human biopsies, we determined mRNA expression of Villin and sucrose isomaltase (*SI*), which are both expressed exclusively in differentiated enterocytes in the villi, and of *c-myc* and cyclin D1 (*CCND1*), both expressed only in the crypts. mRNA expression levels of genes of interest were normalized to hypoxanthine-guanine phosphoribosyltransferase (*HPRT*), which was shown to be the most stable reference gene when analyzed with geNorm [21]. Primers are listed in Table S1.

### Patients and controls and the genetic association study

For the genetic association study, a cohort of 2355 Caucasian IBD patients, consisting of 1162 CD patients and 1193 UC patients was used. This is a subset of a cohort previously described by Weersma and colleagues [22]. Patients were recruited from six University Medical Centers in the Netherlands (details in Table S2). All patients had a confirmed diagnosis of CD or UC, fulfilling standard diagnostic criteria according to clinical, endoscopic, radiological and histopathological findings [23], [24], and were phenotyped according to the Montreal classification [25]. All patients had given written informed consent and all DNA samples and data were handled anonymously. The controls consisted of 853 Dutch blood bank donor controls [4]. All control genotypes were in Hardy-Weinberg equilibrium (data not shown, p>0.05).

### SNP selection and genotyping

Nine tagging single nucleotide polymorphisms (SNPs) to cover the complete *FXR* gene were selected using Haploview 3.32 [26]. Additionally, two functional SNPs, -1G>T and 518T>C (rs56163822 and rs61755050), previously described to affect FXR expression and function [27], respectively, were selected. Two of the tagging SNP assays failed for technical reasons. With the remaining seven tagging SNPs, 89% of the *FXR* gene could be tagged with a genetic variance above 3%. Rs numbers and chromosomal location of the SNPs are shown in Table S3. Genotyping was successful in >98% of all controls, while call rates for all SNPs in patients were >95%, except for rs11110395 (72%) and rs10860603 (91%). From one cohort there was less DNA available so that these SNPs could not be genotyped in that cohort and rs11110395 failed in some cases for technical reasons. Genotyping was performed using TaqMan assays on a TaqMan 7900 HT (Applied Biosystems, Foster City, California, USA). All reported p values are uncorrected unless stated otherwise.

### Statistical analysis

Statistical significance in mRNA expression study was determined by the Student's t-test or the non-parametric Mann-Whitney U test as appropriate. Correlation and regression analyses were used to determine the relationships between expression values. Statistical significance for correlation was determined by Spearman's coefficient test. All statistical calculations were performed with GraphPad PRISM software (Graphpad Software, La Jolla, CA, USA). Two-sided p-values <0.05 are considered statistically significant.

Statistical analysis of the genetic association study was performed using 2-tailed χ^2^ tests of case vs. control allele and haplotype counts for tagging and functional SNPs in Haploview v4.0. [26]. P-values, odds ratios (OR) and 95% confidence intervals (95% CI) are shown. The Bonferroni method was used to correct for multiple testing. All tables show the uncorrected p-values.

## Results

### mRNA expression of FXR and its target gene SHP

**Figure 1:**
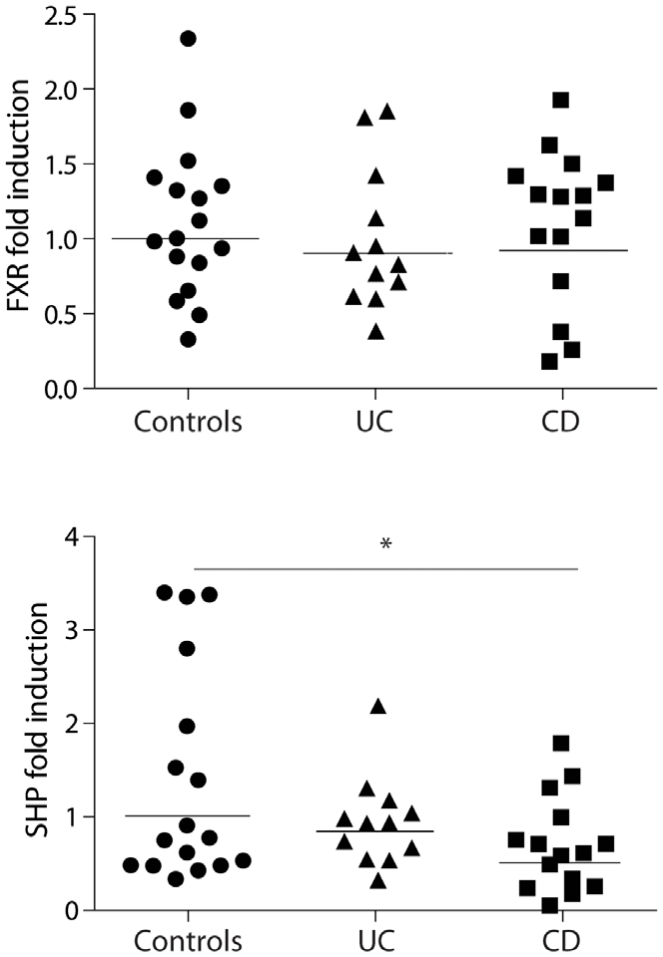
FXR target gene expression is decreased in patients with Crohn's disease. Scatter plot of mRNA expression of *FXR* and *SHP* in the ileal mucosa of healthy controls (circles), ulcerative colitis (triangles) and Crohn's disease patients (squares). Horizontal lines indicate mean values. *p<0.05 compared to healthy controls.

**Figure 2:**
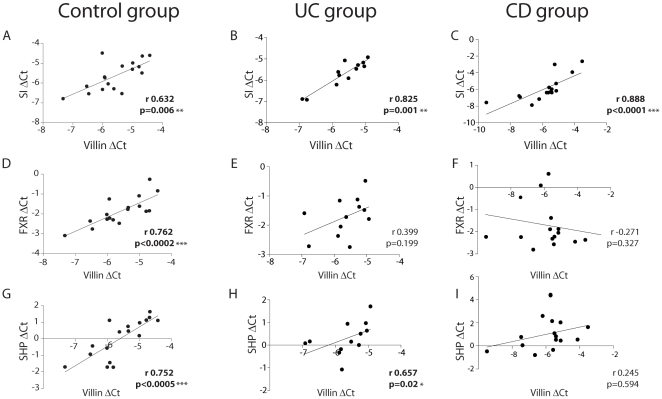
FXR and SHP correlate with Villin in healthy controls but not in Crohn's disease patients. Ileal mRNA expression of *SI*, *FXR* and *SHP* were related by regression analyses to ileal mRNA expression of differentiation marker *Villin* in healthy controls (A, D, G), ulcerative colitis (B, E, H) and Crohn's disease patients (C, F, I). Spearman's coefficients and p values are shown. Values in bold show statistically significant correlations; *p<0.05; **p<0.01, ***p<0.001.

*FXR* and its target gene *SHP* were expressed both in the ileum and ascending colon of IBD patients in remission and controls. Expression levels of *FXR* and *SHP* were markedly lower in the right colon compared to the ileum (53% and 55% lower in the right colon, respectively). There was no significant difference in ileal *FXR* expression between controls, CD and UC patients (Figure 1A). However, ileal expression of *SHP* was 50% lower in CD patients compared to controls (p = 0.039), and 33% lower in UC patients compared to controls (p = 0.21) (Figure 1B). A similar trend, although not significant, was observed in the colon (data not shown). *FXR* and its target genes are exclusively expressed in the differentiated enterocyte in the villi [15], [17], [19]. We, therefore, also correlated *FXR* and *SHP* mRNA expression to *Villin* expression, a marker exclusively expressed in differentiated enterocytes. *Villin* expression was associated with sucrose isomaltase (*SI,* another gene expressed in differentiated enterocytes) expression in controls, UC and CD patients (Figure 2A–C). *Villin* expression correlated also with *FXR* expression in healthy controls. However, the correlation was lost in UC and CD patients (Figure 2D–F). In addition, *Villin* expression showed significant correlation with *SHP* expression in healthy controls and UC patients, whereas the correlation was lost in CD patients (Figure 2G–I). Similar results were found for the correlation between *SI* expression and either *FXR* or *SHP* (data not shown). The expression of the crypt markers *c-myc* and *CCND1* were significantly correlated. However, *c-myc* and *CCDN1* did not correlate to *Villin*, *SI*, *FXR* or *SHP* expression in any of the groups (data not shown).

### Assessment of FXR genetic variation in IBD patients

A total of 2355 IBD patients and 853 controls were genotyped with seven tagging SNPs and two functional SNPs in *FXR*. None of the functional SNPs was associated with the presence of IBD. One of the tagging SNPs, however, displayed a significant association with IBD (rs12313471, p = 0.03, OR 1.32, 95% CI 1.02–1.71; Table S4). CD (n = 1162) and UC patients (n = 1193) were also separately compared to the 853 healthy controls. The same tagging SNP (rs12313471) was associated with UC (p = 0.049, OR 1.32, 95% CI 1.00–1.76; Table S5). None of the SNPs was associated with CD (Table S6). None of the above described associations remained significant after Bonferroni correction for multiple testing.

### Subgroup analyses

Phenotypic information on the localization of the disease was present for 1132 of 1162 (97.4%) patients with CD. We analyzed whether polymorphisms of *FXR* were associated with CD location using the Montreal classification [25]. Patients with L1 (terminal ileum location; n = 257), L2 (colonic location; n = 295) and L3 (ileocolonic location; n = 580) were compared to CD patients with other disease locations. Two tagging SNPs displayed a significant association with ileal CD (L1; rs11110390, p = 0.03, OR 1.26, 95% CI 1.02–1.55 and rs4764980, p = 0.03, OR 0.80, 95% CI 0.65–0.98, Table S7). None of the SNPs was associated with colonic CD (L2, Table S8). Two SNPs showed a significant association with ileocolonic CD (L3), namely the functional SNP 518T>C (p = 0.015, OR 3.08, 95% CI 1.08–8.83) and one of the tagging SNPs (rs10860603, p = 0.013, OR 1.39, 95% CI 1.07–1.81; Table S9). None of these subgroup analyses, however, remained significant after Bonferroni correction for multiple testing.

## Discussion

Although the exact etiology of IBD is not completely understood, several lines of evidence point to an impaired intestinal barrier function and an abnormal immune response in genetically susceptible hosts. Recently, we reported that activation of the nuclear receptor FXR prevents inflammation in animal models of IBD with improvement of colitis symptoms, preservation of the intestinal epithelial barrier function and reduction of goblet cell loss [17]. Furthermore, a negative cross-talk between FXR and the inflammatory response at the intestinal level was demonstrated [19], probably contributing to an attenuated intestinal inflammatory status. In the present study, we showed that ileal mRNA expression of the FXR target gene *SHP* is markedly reduced in Crohn's colitis patients, whereas *FXR* expression remained unchanged. This suggests that FXR activity is decreased in this IBD subtype. Previously published genome-wide association scans in IBD patients did not identify loci containing the *FXR* gene [9], [10]. Since these association studies explain only a small part of the genetic contribution in IBD, we took a candidate-gene approach and studied genetic variation in *FXR*. In the present study, none of the functional or common tagging SNPs proved to be significantly associated with CD or UC. Interestingly, the SNP 518T>C, resulting in the amino acid change M173T, showed an association with the ileocolonic phenotype of CD (Montreal L3)(25) (p = 0.015, OR 3.08, 95% CI 1.08–8.83). The same allele has previously been shown to be associated with intrahepatic cholestasis of pregnancy [27], and to result in a 60% decrease in transcriptional activity of FXR. Although the M173T was not significantly associated with CD after correction for multiple testing, it may well be that it plays a modifier role in the etiology of CD in conjunction with other genes. In addition, other weak associations of different tagging SNPs with colonic or ileocolonic phenotypes disappeared after correction for multiple testing. Thus, a primary genetic defect underlying the role of FXR in CD could not be substantiated. Since the functional SNP 518T>C has a very low prevalence, the possibility of a type II error cannot be excluded. Moreover, two of the selected tagging SNP assays failed due to technical reasons. Thus it cannot be excluded that some common SNPs tagging in the remaining 11% of the *FXR* gene display an association with IBD.

Also other explanations accounting for the decreased FXR activity in CD should be considered. This includes the possibility that bile salt uptake in the ileum is reduced, for example due to decreased intestinal transit times. Indeed, several studies have shown increased fecal excretion of bile salts in patients with CD in clinical remission [28]–[31].

Another mechanism contributing to this phenomenon could be an intrinsically different regulation of bile salt uptake in the ileum in CD patients [32].

Lastly, reduced FXR target gene expression may be secondary to the reciprocal inhibition of FXR by NF-κB [19], [20]. It is well established that a range of pro-inflammatory cytokines is upregulated in the mucosa of IBD patients in remission, potentially resulting in downregulated FXR activity, leading to the observed reduced *SHP* expression in the current study [33].

In conclusion, we found that *FXR* expression in the ileum is altered in patients with Crohn's colitis. This could not be explained by the presence of common SNPs in the *FXR* gene. Treatment with synthetic FXR agonists may overcome the decrease in FXR activation, possibly resulting in an amelioration of ileocolitis in patients with CD.

## Supporting Information

Table S1 **qRT-PCR primer list.**
(DOC)

Table S2 **Number of patients and hospitals.**
(DOC)

Table S3 **RS numbers and chromosomal locations of the SNPs in FXR.**
(DOC)

Table S4 **Association of genetic variants in FXR with the entire IBD cohort (patients with Crohn's disease and ulcerative colitis).**
(DOC)

Table S5 **Association of genetic variants in FXR with ulcerative colitis.**
(DOC)

Table S6 **Association of genetic variants in FXR with Crohn's disease.**
(DOC)

Table S7 **Association of genetic variants in FXR: subgroup analysis of patients with L1 Crohn's disease vs. Crohn's disease with other disease localization.**
(DOC)

Table S8 **Association of genetic variants in FXR: subgroup analysis of patients with L2 Crohn's disease vs. Crohn's disease with other disease localization.**
(DOC)

Table S9 **Association of genetic variants in FXR: subgroup analysis of patients with L3 Crohn's disease vs. Crohn's disease with other disease localization.**
(DOC)
