# Supplementary material for: Farnesoid X Receptor (FXR) Activation and FXR Genetic Variation in Inflammatory Bowel Disease
Source: PLoS One. 2011 Aug 22;6(8):e23745. doi: 10.1371/journal.pone.0023745 (PMC3161760; doi:10.1371/journal.pone.0023745)
Supplement: Table S1 — qRT-PCR primer list. (DOC) [file pone.0023745.s001.doc]

**Supplementary Table S1. qRT-PCR primer list.**

| **FXR F** | 5'-CTACCAGGATTTCAGACTTTGGAC-3' |
| --- | --- |
| **FXR R** | 5'-GAACATAGCTTCAACCGCAGAC-3' |
| **SHP F** | 5'-AGGGACCATCCTCTTCAACC-3' |
| **SHP R** | 5'-TTCACACAGCACCCAGTGAG-3' |
| **HRPT F** | 5'-ATTGTAATGACCAGTCAACAGGG-3' |
| **HRPT R** | 5'-GCATTGTTTTGCCAGTGTCAA-3' |
| **VILLIN F** | 5'-AGGGCAAGAGGAACGTGGT-3' |
| **VILLIN R** | 5'-TCCCCTCGGTTGAAACTCTTC-3' |
| **SI F** | 5'-GGAGATACACCAGAACAAGTAGTTCAA-3' |
| **SI R** | 5'-AATCCAAGATTCCAATATGCTGG-3' |
| **c-myc F** | 5'-CCACCACCAGCAGCGACT-3' |
| **c-myc R** | 5'-CAGAAACAACATCGATTTCTTCCTC-3' |
| **CCDN1 F** | 5'-CGTGGCCTCTAAGATGAAGGA-3' |
| **CCDN1 R** | 5'-CGGTGTAGATGCACAGCTTCT-3' |
