# Supplementary material for: Farnesoid X Receptor (FXR) Activation and FXR Genetic Variation in Inflammatory Bowel Disease
Source: PLoS One. 2011 Aug 22;6(8):e23745. doi: 10.1371/journal.pone.0023745 (PMC3161760; doi:10.1371/journal.pone.0023745)
Supplement: Table S2 — Number of patients and hospitals. (DOC) [file pone.0023745.s002.doc]

**Supplementary Table S2. Number of patients and hospitals.**

| **Hospital** | **Patients** |
| --- | --- |
| Academic Medical Centre Amsterdam | 439 |
| VU University Medical Centre Amsterdam | 647 |
| University Medical Centre Groningen | 547 |
| University Medical Centre Leiden | 494 |
| University Medical Centre St. Radboud, Nijmegen | 148 |
| University Medical Centre Utrecht | 80 |
| **Total number of patients** | **2355** |
