# Supplementary material for: Farnesoid X Receptor (FXR) Activation and FXR Genetic Variation in Inflammatory Bowel Disease
Source: PLoS One. 2011 Aug 22;6(8):e23745. doi: 10.1371/journal.pone.0023745 (PMC3161760; doi:10.1371/journal.pone.0023745)
Supplement: Table S3 — RS numbers and chromosomal locations of the SNPs in FXR. (DOC) [file pone.0023745.s003.doc]

**Supplementary Table S3. RS numbers and chromosomal locations of the SNPs in FXR.**

|  | **SNP number** | **Chromosomal location on chromosome 12 (dbSNP build 132)** |
| --- | --- | --- |
| **Tagging SNPs** | rs11837065^*^ | 100859733 |
|  | rs12313471 | 100864393 |
|  | rs11110390 | 100874901 |
|  | rs4764980 | 100885107 |
|  | rs11110395 | 100888664 |
|  | rs17030285^*^ | 100929963 |
|  | rs11610264 | 100932375 |
|  | rs10860603 | 100943948 |
|  | rs35739 | 100948515 |
| **Functional SNPs** | -1G>T^#^ | 100887351 |
|  | 518T>C^#^ | 100926058 |

^*^The rs11837065 and rs17030285 SNPs failed for technical reasons.

^#^The rs numbers of the functional SNPs are: rs56163822 (-1G>T) and rs61755050 (518T>C).
