# Supplementary material for: Farnesoid X Receptor (FXR) Activation and FXR Genetic Variation in Inflammatory Bowel Disease
Source: PLoS One. 2011 Aug 22;6(8):e23745. doi: 10.1371/journal.pone.0023745 (PMC3161760; doi:10.1371/journal.pone.0023745)
Supplement: Table S4 — Association of genetic variants in FXR with the entire IBD cohort (patients with Crohn's disease and ulcerative colitis). (DOC) [file pone.0023745.s004.doc]

**Supplementary Table S4. Association of genetic variants in FXR with the entire IBD cohort (patients with Crohn’s disease and ulcerative colitis).**

|  |  | **IBD patients** | | | **Controls** | | | **p value^*^** | **OR** | **95% CI** |
| --- | --- | --- | --- | --- | --- | --- | --- | --- | --- | --- |
|  |  | Allele counts | |  | Allele counts | |  |  |  |  |
|  |  | Minor | Major | MAF | Minor | Major | MAF |  |  |  |
| -1G>T | A/C^#^ | 125 | 4461 | 0.027 | 36 | 1588 | 0.022 | 0.2674 | 1.21 | 0.84-1.76 |
| 518T>C | G/A | 29 | 4545 | 0.006 | 6 | 1616 | 0.004 | 0.2227 | 1.52 | 0.65-3.57 |
| rs12313471 | G/A | 277 | 4245 | 0.061 | 76 | 1548 | 0.047 | **0.0317** | 1.32 | 1.02-1.71 |
| rs11110390 | T/C | 1473 | 3103 | 0.322 | 544 | 1070 | 0.337 | 0.2641 | 0.93 | 0.83-1.05 |
| rs4764980 | A/G | 2271 | 2261 | 0.501 | 778 | 832 | 0.517 | 0.2179 | 1.07 | 0.96-1.20 |
| rs11110395 | T/G | 156 | 3182 | 0.047 | 84 | 1538 | 0.052 | 0.4365 | 0.89 | 0.68-1.17 |
| rs11610264 | C/T | 1343 | 3141 | 0.300 | 458 | 1160 | 0.283 | 0.2138 | 1.08 | 0.95-1.23 |
| rs10860603 | A/G | 549 | 3687 | 0.130 | 214 | 1398 | 0.133 | 0.7492 | 0.97 | 0.82-1.15 |
| rs35739 | C/T | 2033 | 2441 | 0.454 | 712 | 900 | 0.442 | 0.3790 | 1.05 | 0.94-1.18 |

OR = odds ratio; 95% CI = 95% confidence interval.

^#^ Minor allele / major allele; MAF = minor allele frequency

^*^ Two-tailed P values were calculated by χ^2^ analysis of allele counts

Significant p values are shown in bold.
