# Supplementary material for: Farnesoid X Receptor (FXR) Activation and FXR Genetic Variation in Inflammatory Bowel Disease
Source: PLoS One. 2011 Aug 22;6(8):e23745. doi: 10.1371/journal.pone.0023745 (PMC3161760; doi:10.1371/journal.pone.0023745)
Supplement: Table S5 — Association of genetic variants in FXR with ulcerative colitis. (DOC) [file pone.0023745.s005.doc]

**Supplementary Table S5. Association of genetic variants in FXR with ulcerative colitis.**

|  |  | **UC patients** | | | **Controls** | | | **p value^*^** | **OR** | **95% CI** |
| --- | --- | --- | --- | --- | --- | --- | --- | --- | --- | --- |
|  |  | Allele counts | |  | Allele counts | |  |  |  |  |
|  |  | Minor | Major | MAF | Minor | Major | MAF |  |  |  |
| -1G>T | A/C^#^ | 58 | 2264 | 0.025 | 36 | 1588 | 0.022 | 0.5688 | 1.12 | 0.74-1.70 |
| 518T>C | G/A | 10 | 2292 | 0.004 | 6 | 1616 | 0.004 | 0.7549 | 1.11 | 0.42-2.95 |
| rs12313471 | G/A | 141 | 2155 | 0.061 | 76 | 1548 | 0.047 | **0.0487** | 1.32 | 1.00-1.76 |
| rs11110390 | T/C | 719 | 1605 | 0.309 | 544 | 1070 | 0.337 | 0.0673 | 0.88 | 0.77-1.01 |
| rs4764980 | A/G | 1182 | 1116 | 0.514 | 778 | 832 | 0.483 | 0.0554 | 1.13 | 1.00-1.29 |
| rs11110395 | T/G | 58 | 1340 | 0.041 | 84 | 1538 | 0.052 | 0.1824 | 0.80 | 0.57-1.12 |
| rs11610264 | C/T | 705 | 1585 | 0.308 | 458 | 1160 | 0.283 | 0.0949 | 1.13 | 0.98-1.29 |
| rs10860603 | A/G | 283 | 1769 | 0.138 | 214 | 1398 | 0.133 | 0.6507 | 1.04 | 0.86-1.26 |
| rs35739 | C/T | 1053 | 1227 | 0.462 | 712 | 900 | 0.442 | 0.2135 | 1.08 | 0.95-1.23 |

OR = odds ratio; 95% CI = 95% confidence interval

^#^ Minor allele / major allele; MAF = minor allele frequency

^*^ Two-tailed P values were calculated by χ^2^ analysis of allele counts

Significant p values are shown in bold.
