# Supplementary material for: Farnesoid X Receptor (FXR) Activation and FXR Genetic Variation in Inflammatory Bowel Disease
Source: PLoS One. 2011 Aug 22;6(8):e23745. doi: 10.1371/journal.pone.0023745 (PMC3161760; doi:10.1371/journal.pone.0023745)
Supplement: Table S6 — Association of genetic variants in FXR with Crohn's disease. (DOC) [file pone.0023745.s006.doc]

**Supplementary Table S6. Association of genetic variants in FXR with Crohn’s disease.**

|  |  | **CD patients** | | | **Controls** | | | **p value^*^** | **OR** | **95% CI** |
| --- | --- | --- | --- | --- | --- | --- | --- | --- | --- | --- |
|  |  | Allele counts | |  | Allele counts | |  |  |  |  |
|  |  | Minor | Major | MAF | Minor | Major | MAF |  |  |  |
| -1G>T | A/C^#^ | 67 | 2197 | 0.030 | 36 | 1588 | 0.022 | 0.1550 | 1.33 | 0.88-2.00 |
| 518T>C | G/A | 19 | 2253 | 0.008 | 6 | 1616 | 0.004 | 0.0725 | 2.05 | 0.84-4.99 |
| rs12313471 | G/A | 136 | 2090 | 0.061 | 76 | 1548 | 0.047 | 0.0548 | 1.32 | 0.99-1.76 |
| rs11110390 | T/C | 754 | 1498 | 0.335 | 544 | 1070 | 0.337 | 0.8845 | 1.01 | 0.88-1.16 |
| rs4764980 | A/G | 1089 | 1145 | 0.487 | 778 | 832 | 0.483 | 0.7954 | 1.02 | 0.89-1.16 |
| rs11110395 | T/G | 98 | 1842 | 0.051 | 84 | 1538 | 0.052 | 0.8636 | 1.03 | 0.72-1.31 |
| rs11610264 | C/T | 638 | 1556 | 0.291 | 458 | 1160 | 0.283 | 0.6024 | 1.04 | 0.90-1.20 |
| rs10860603 | A/G | 266 | 1918 | 0.122 | 214 | 1398 | 0.133 | 0.3153 | 0.91 | 0.75-1.10 |
| rs35739 | C/T | 980 | 1214 | 0.447 | 712 | 900 | 0.442 | 0.7597 | 1.02 | 0.90-1.16 |

OR = odds ratio; 95% CI = 95% confidence interval

^#^ Minor allele / major allele; MAF = minor allele frequency

^*^ Two-tailed P values were calculated by χ^2^ analysis of allele counts
