# Supplementary material for: Farnesoid X Receptor (FXR) Activation and FXR Genetic Variation in Inflammatory Bowel Disease
Source: PLoS One. 2011 Aug 22;6(8):e23745. doi: 10.1371/journal.pone.0023745 (PMC3161760; doi:10.1371/journal.pone.0023745)
Supplement: Table S7 — Association of genetic variants in FXR: subgroup analysis of patients with L1 Crohn's disease vs. Crohn's disease with other disease localization. (DOC) [file pone.0023745.s007.doc]

**Supplementary Table S7. Association of genetic variants in FXR: subgroup analysis of patients with L1 Crohn’s disease vs. Crohn’s disease with other disease localization.**

|  |  | **CD L1 patients** | | | **CD patients** | | | **p value^*^** | **OR** | **95% CI** |
| --- | --- | --- | --- | --- | --- | --- | --- | --- | --- | --- |
|  |  | Allele counts | |  | Allele counts | |  |  |  |  |
|  |  | Minor | Major | MAF | Minor | Major | MAF |  |  |  |
| -1G>T | A/C^#^ | 15 | 483 | 0.030 | 49 | 1657 | 0.029 | 0.8701 | 1.10 | 0.61-1.96 |
| 518T>C | G/A | 1 | 503 | 0.002 | 18 | 1690 | 0.011 | 0.0674 | 0.35 | 0.07-1.87 |
| rs12313471 | G/A | 26 | 462 | 0.053 | 105 | 1575 | 0.062 | 0.4517 | 0.87 | 0.56-1.34 |
| rs11110390 | T/C | 187 | 309 | 0.377 | 551 | 1147 | 0.324 | **0.0294** | 1.26 | 1.02-1.55 |
| rs4764980 | A/G | 221 | 279 | 0.442 | 834 | 840 | 0.498 | **0.0273** | 0.80 | 0.65-0.98 |
| rs11110395 | T/G | 26 | 400 | 0.061 | 71 | 1393 | 0.048 | 0.3021 | 1.30 | 0.82-2.06 |
| rs11610264 | C/T | 138 | 348 | 0.284 | 486 | 1166 | 0.294 | 0.6625 | 0.95 | 0.76-1.19 |
| rs10860603 | A/G | 48 | 436 | 0.099 | 211 | 1431 | 0.129 | 0.0830 | 0.76 | 0.54-1.05 |
| rs35739 | C/T | 211 | 273 | 0.436 | 754 | 896 | 0.457 | 0.4139 | 0.92 | 0.75-1.13 |

OR = odds ratio; 95% CI = 95% confidence interval

^#^ Minor allele / major allele; MAF = minor allele frequency

^*^ Two-tailed p values were calculated by χ^2^ analysis of allele counts

Significant p values are shown in bold.
