# Supplementary material for: Farnesoid X Receptor (FXR) Activation and FXR Genetic Variation in Inflammatory Bowel Disease
Source: PLoS One. 2011 Aug 22;6(8):e23745. doi: 10.1371/journal.pone.0023745 (PMC3161760; doi:10.1371/journal.pone.0023745)
Supplement: Table S8 — Association of genetic variants in FXR: subgroup analysis of patients with L2 Crohn's disease vs. Crohn's disease with other disease localization. (DOC) [file pone.0023745.s008.doc]

**Supplementary Table S8. Association of genetic variants in FXR: subgroup analysis of patients with L2 Crohn’s disease vs. Crohn’s disease with other disease localization.**

|  |  | **CD L2 patients** | | | **CD patients** | | | **p value^*^** | **OR** | **95% CI** |
| --- | --- | --- | --- | --- | --- | --- | --- | --- | --- | --- |
|  |  | Allele counts | |  | Allele counts | |  |  |  |  |
|  |  | Minor | Major | MAF | Minor | Major | MAF |  |  |  |
| -1G>T | A/C^#^ | 17 | 561 | 0.029 | 47 | 1579 | 0.029 | 0.9503 | 1.05 | 0.60-1.84 |
| 518T>C | G/A | 3 | 573 | 0.005 | 16 | 1620 | 0.010 | 0.3065 | 0.66 | 0.21-2.11 |
| rs12313471 | G/A | 28 | 538 | 0.049 | 103 | 1499 | 0.064 | 0.2032 | 0.77 | 0.51-1.19 |
| rs11110390 | T/C | 184 | 388 | 0.322 | 554 | 1068 | 0.342 | 0.3870 | 0.92 | 0.75-1.12 |
| rs4764980 | A/G | 275 | 293 | 0.484 | 780 | 826 | 0.486 | 0.9502 | 0.99 | 0.82-1.20 |
| rs11110395 | T/G | 30 | 472 | 0.060 | 67 | 1321 | 0.048 | 0.3174 | 1.27 | 0.82-1.98 |
| rs11610264 | C/T | 173 | 395 | 0.305 | 451 | 1119 | 0.287 | 0.4366 | 1.09 | 0.88-1.34 |
| rs10860603 | A/G | 59 | 489 | 0.108 | 200 | 1378 | 0.127 | 0.2394 | 0.84 | 0.62-1.14 |
| rs35739 | C/T | 258 | 304 | 0.459 | 707 | 865 | 0.450 | 0.7029 | 1.04 | 0.86-1.26 |

OR = odds ratio; 95% CI = 95% confidence interval

^#^ Minor allele / major allele; MAF = minor allele frequency

Two-tailed P values were calculated by χ^2^ analysis of allele counts
