# Supplementary material for: Farnesoid X Receptor (FXR) Activation and FXR Genetic Variation in Inflammatory Bowel Disease
Source: PLoS One. 2011 Aug 22;6(8):e23745. doi: 10.1371/journal.pone.0023745 (PMC3161760; doi:10.1371/journal.pone.0023745)
Supplement: Table S9 — Association of genetic variants in FXR: subgroup analysis of patients with L3 Crohn's disease vs. Crohn's disease with other disease localization. (DOC) [file pone.0023745.s009.doc]

**Supplementary Table S9. Association of genetic variants in FXR: subgroup analysis of patients with L3 Crohn’s disease vs. Crohn’s disease with other disease localization.**

|  |  | **CD L3 patients** | | | **CD patients** | | | **p value^*^** | **OR** | **95% CI** |
| --- | --- | --- | --- | --- | --- | --- | --- | --- | --- | --- |
|  |  | Allele counts | |  | Allele counts | |  |  |  |  |
|  |  | Minor | Major | MAF | Minor | Major | MAF |  |  |  |
| -1G>T | A/C^#^ | 32 | 1096 | 0.028 | 32 | 1044 | 0.030 | 0.8481 | 0.95 | 0.58-1.56 |
| 518T>C | G/A | 15 | 1117 | 0.013 | 4 | 1076 | 0.004 | **0.0150** | 3.08 | 1.08-8.83 |
| rs12313471 | G/A | 77 | 1037 | 0.069 | 54 | 1000 | 0.051 | 0.0806 | 1.37 | 0.96-1.95 |
| rs11110390 | T/C | 367 | 759 | 0.326 | 371 | 697 | 0.347 | 0.2879 | 0.91 | 0.76-1.08 |
| rs4764980 | A/G | 559 | 547 | 0.505 | 496 | 572 | 0.464 | 0.0558 | 1.18 | 1.00-1.39 |
| rs11110395 | T/G | 41 | 921 | 0.043 | 56 | 872 | 0.060 | 0.0808 | 0.70 | 0.46-1.05 |
| rs11610264 | C/T | 313 | 771 | 0.289 | 311 | 743 | 0.295 | 0.7479 | 0.97 | 0.80-1.17 |
| rs10860603 | A/G | 152 | 942 | 0.139 | 107 | 925 | 0.104 | **0.0130** | 1.39 | 1.07-1.81 |
| rs35739 | C/T | 496 | 592 | 0.456 | 469 | 577 | 0.448 | 0.7276 | 1.03 | 0.87-1.22 |

(rs10860603, p=0.01, OR 1.39, 95% CI 1.07-1.81; Supplementary Table 9).

OR = odds ratio; 95% CI = 95% confidence interval

^#^ Minor allele / major allele; MAF = minor allele frequency

^*^ Two-tailed P values were calculated by χ^2^ analysis of allele counts

Significant p values are shown in bold.
